# Applicability of Anatomical Landmarks for Chest Compression Depth in Cardiopulmonary Resuscitation for Children

**DOI:** 10.1038/s41598-020-58649-5

**Published:** 2020-02-05

**Authors:** Yong Hwan Kim, Jun Ho Lee, Dong Woo Lee, Yun Gyu Song, Kyoung Yul Lee, Young Hwan Lee, Seong Youn Hwang, Seok Ran Yeom

**Affiliations:** 1 Department of Emergency Medicine, Samsung Changwon Hospital, Sungkyunkwan University School of Medicine, Changwon, South Korea; 2 Department of Radiology, Samsung Changwon Hospital, Sungkyunkwan University School of Medicine, Changwon, South Korea; 3 Department of Physical Education, Kyungnam University, Changwon, South Korea; 4 Department of Emergency Medicine, College of Medicine, Soonchunhyang University Bucheon Hospital, Bucheon, South Korea; 5 Department of Emergency Medicine, Pusan National University, School of Medicine, Pusan National University Hospital, Busan, South Korea

**Keywords:** Paediatric research, Paediatric research

## Abstract

We evaluated the applicability of the neck and sternal notch (SN) as anatomical landmarks for paediatric chest compression (CC) depth using chest computed tomography. The external anteroposterior diameter (EAPD) of the neck and chest at the SN level, mid-point between two landmarks (mid-landmark), and EAPD of the chest at the lower half of the sternum (EDLH) were measured. To estimate the depths of the landmarks from a virtual point at the same height as the position for CC, we calculated the differences between the EAPDs of the neck, SN, mid-landmark, and EDLH. We analysed the relationship between the depths of the landmarks and one-third EDLH using Bland–Altman plots. In all, 506 paediatric patients aged 1–9 years were enrolled. The depths of the neck, SN, and mid-landmark were 53.7 ± 10.0, 37.8 ± 8.5, and 45.8 ± 9.0 mm, respectively. The mean one-third EDLH was 46.8 ± 7.0 mm. The means of the differences between the depths of the neck and one-third EDLH, depths of the SN and one-third EDLH, and depths of the mid-landmark and one-third EDLH were 9.0, −6.9, and 1.0 mm, respectively. The SN and neck are inappropriate landmarks to guide compression depth in paediatric CPR.

## Introduction

Current cardiopulmonary resuscitation (CPR) guidelines emphasise the importance of chest compressions (CCs). CCs are important not only for adults but also for infants and children, who have relatively high rates of respiratory arrest^1^. The American Heart Association (AHA) and European Resuscitation Council (ERC) recommends a CC depth of at least one-third of the chest anteroposterior (AP) dimension in children^1,2^.

Given the characteristics of children whose body sizes vary greatly with age, different CC depths may be required for them during CPR. Because of this variability, it may be difficult to meet the recommended criterion for fractional CC depths in actual child CPR situations. In line with these concerns, a previous study observed that the target CC depth recommended by the guidelines was not achieved in 81% of CPR events^3^. Identifying an anatomical landmark close to one-third of the child’s chest dimension may increase the probability of achieving the target CC depth during CPR. However, studies on anatomical landmarks of CC depth are limited.

In a recent study using chest computed tomography (CT) in adults, Kim *et al*. reported that the suprasternal notch could be an anatomical landmark corresponding to one-fourth of the AP diameter of the chest^4^. However, in that study, one-fourth was less than one-third fractional CC depth recommended for children. Additionally, the thorax grows and develops from birth to adolescence. The ratio of the vertical diameter to the transverse diameter of the chest varies from approximately 1:1 in infants to approximately 1:1.5 in adults^5^. Considering the difference in the aspect ratio between adults and children, the landmark for CC depth in children is likely to be deeper than the sternal notch, which is the landmark identified in adults. In this context, we present the neck as a landmark for CC depth for children, which is slightly deeper than the sternal notch in the supine position.

The purpose of this study was to evaluate the feasibility of the neck and sternal notch as anatomical landmarks for paediatric CC depth using chest CT.

## Results

### Demographics and measured variables according to age group

Of the 531 paediatric patients who underwent chest CT scans during the study period, 25 with pectus excavatum (15), pigeon chest (1), other chest deformities (2), poor CT image (2), large amount of subcutaneous emphysema (3), and skin inflammation around the study landmark (2) were excluded. Of the 506 included patients, 298 (58.9%) were men. The mean age of the paediatric patients was 62 months. The patients’ demographic data and average external AP diameter measured at each point are shown in Table 1.

**Table 1:** Demographics and measured variables according to age group.

Age (months)/Variable	Total (n = 506)	13–24 (n = 73)	25–36 (n = 67)	37–48 (n = 49)	49–60 (n = 56)	61–72 (n = 57)	73–84 (n = 55)	85–96 (n = 56)	97–108 (n = 48)	109–119 (n = 45)	p-value
Male, n (%)	298 (58.9)	47 (64.4)	37 (55.2)	27 (55.1)	33 (58.9)	30 (52.6)	35 (63.6)	34 (60.7)	28 (58.3)	27 (60)	<0.001
EDN, mm	86.7 ± 12.8	73.8 ± 5.7	76.1 ± 5.0	80.5 ± 6.3	84.7 ± 7.0	86.7 ± 8.0	90.7 ± 8.1	93.0 ± 9.0	100.4 ± 12.0	105.0 ± 12.0	<0.001
EDML, mm	94. ± 13.8	81.0 ± 5.6	83.8 ± 4.9	87.5 ± 6.0	91.8 ± 7.4	94.5 ± 8.7	98.1 ± 8.3	101.3 ± 9.7	109.9 ± 13.7	115.2 ± 13.4	<0.001
EDSN, mm	102.6 ± 15.1	88.3 ± 6.2	91.5 ± 5.3	94.6 ± 6.1	99.0 ± 8.3	102.3 ± 9.9	105.5 ± 9.0	109.7 ± 10.9	119.4 ± 15.7	125.4 ± 15.3	<0.001
EDLH, mm	140.4 ± 30.0	116.8 ± 8.4	123.5 ± 7.5	129.5 ± 9.3	136.5 ± 9.3	140.2 ± 11.4	148.0 ± 11.6	151.9 ± 13.4	164.1 ± 19.3	172.2 ± 17.5	<0.001
Neck depth, mm	53.7 ± 10.0	43.0 ± 5.7	47.4 ± 5.1	49.0 ± 5.7	51.8 ± 5.1	53.4 ± 7.0	57.3 ± 7.1	58.9 ± 6.9	63.7 ± 10.1	67.2 ± 8.9	<0.001
Mid-landmark depth, mm	45.8 ± 9.0	35.8 ± 4.9	39.7 ± 5.0	42.0 ± 5.3	44.7 ± 4.5	45.6 ± 6.3	49.9 ± 6.3	50.6 ± 6.6	54.2 ± 8.8	57.0 ± 7.8	<0.001
SN depth, mm	37.8 ± 8.5	28.6 ± 4.9	32.0 ± 5.4	34.9 ± 5.5	37.5 ± 4.8	37.8 ± 6.4	42.5 ± 6.3	42.2 ± 7.1	44.7 ± 8.4	46.7 ± 7.8	<0.001
1/3 EDLH, mm	46.8 ± 7.0	38.9 ± 2.8	41.2 ± 2.5	43.2 ± 3.1	45.5 ± 3.1	46.7 ± 3.8	49.3 ± 3.9	50.6 ± 4.5	54.7 ± 6.4	57.4 ± 5.8	<0.001
1/3 EDLH > neck depth, n (%)	26 (5.1)	6 (8.2)	2 (3.0)	3 (6.1)	3 (5.4)	3 (5.3)	1 (1.8)	2 (3.6)	3 (6.3)	3 (6.7)	0.699
1/3 EDLH < SN depth, n (%)	13 (2.6)	0 (0)	1 (1.5)	1 (2.0)	1 (1.8)	1 (1.8)	3 (5.5)	3 (5.4)	3 (6.3)	0 (0)	0.257

n: number, SN: sternal notch, EDN: external anteroposterior diameter of the neck, EDML: external anteroposterior diameter of the chest at the mid-point between the two landmarks, EDSN: external anteroposterior diameter of the chest at the sternal notch, EDLH: external anteroposterior diameter of the chest at the lower half of the sternum.

The depth of each landmark is shown in Table 1 (neck, 53.7 ± 10.0 mm; SN, 37.8 ± 8.5 mm; and mid-landmark, 45.8 ± 9.0 mm). The mean one-third of the external AP diameter at the lower half of the sternum was 46.8 ± 7 mm. One-sample *t*-test indicated a significant difference between the depth of each landmark and one-third AP diameter of the chest (all P values < 0.001).

### Bland–Altman plot of the degree of agreement between the depth of each landmark and one-third external anteroposterior diameter at the lower half of the sternum

**Figure 1:**
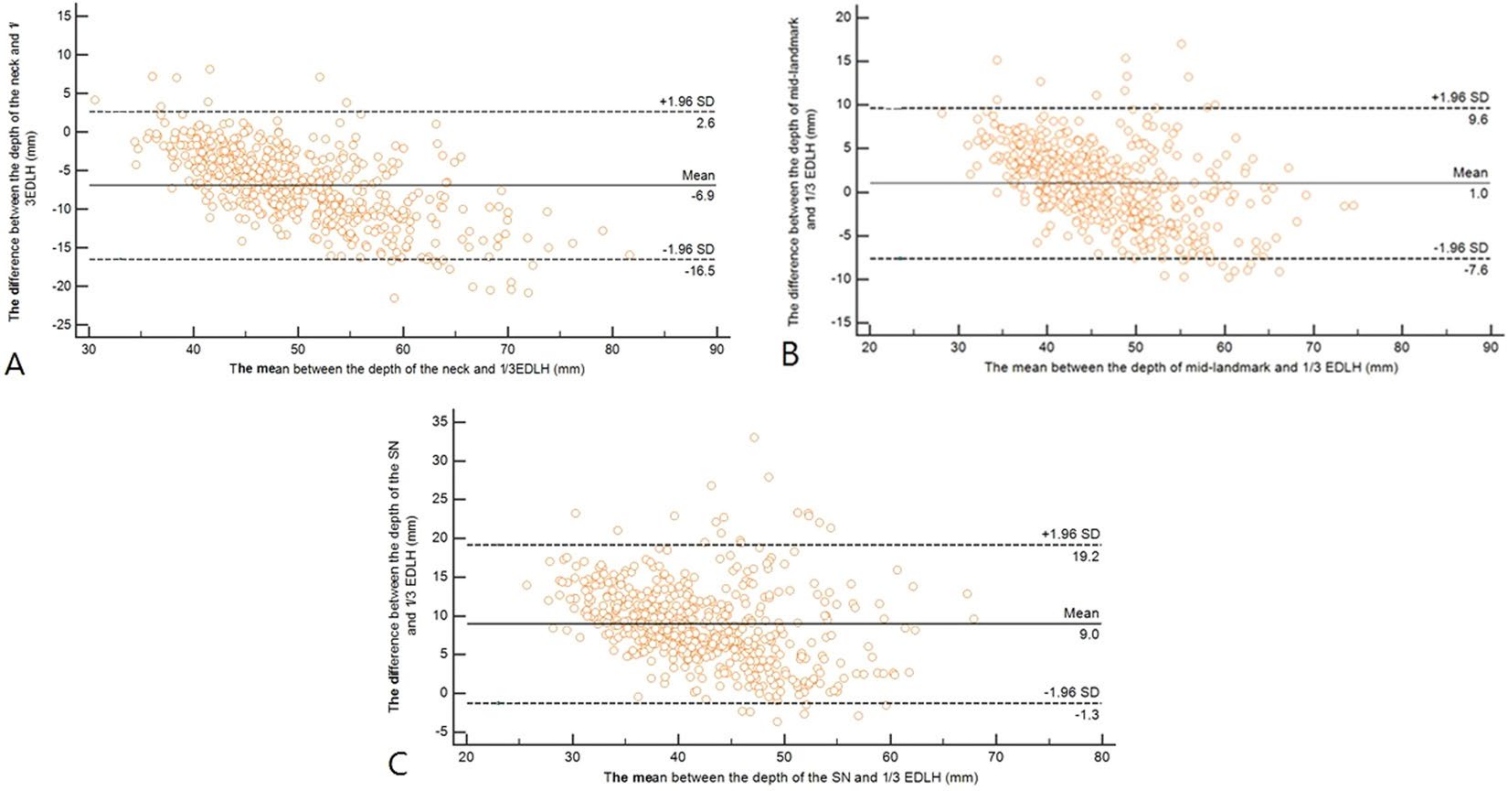
Bland–Altman plot of the degree of agreement between the depth of each landmark and one-third anteroposterior diameter at the lower half level of the sternum. SD: standard deviation, SN: sternal notch, EDLH: external anteroposterior diameter of the chest at the lower half of the sternum.

Bland–Altman plots demonstrating the differences between the depths of each landmark and one-third external AP diameter at the lower half of the sternum are illustrated in Fig. 1. The mean differences between the depth of each landmark and one-third external AP diameter at the lower half of the sternum were as follows: sternal notch vs. one-third external AP diameter at the lower half of the sternum = 9.0 mm, neck vs. one-third external AP diameter at the lower half of the sternum = −6.9 mm, and mid-landmark vs. one-third external AP diameter at the lower half of the sternum = 1.0 mm.

### Relative location corresponding to one-third external anteroposterior diameter at the lower half of the sternum between the neck and the sternal notch

The overall proportion of patients whose one-third external AP diameter at the lower half of the sternum was deeper than the depth of the neck was 5.1% (26/506), and no statistical difference was found between the different age groups. The overall proportion of patients whose one-third external AP diameter at the lower half of the sternum was shallower than the depth of the sternal notch was 2.6% (13/506), and no statistical difference was found between the different age groups (Table 1).

**Figure 2:**
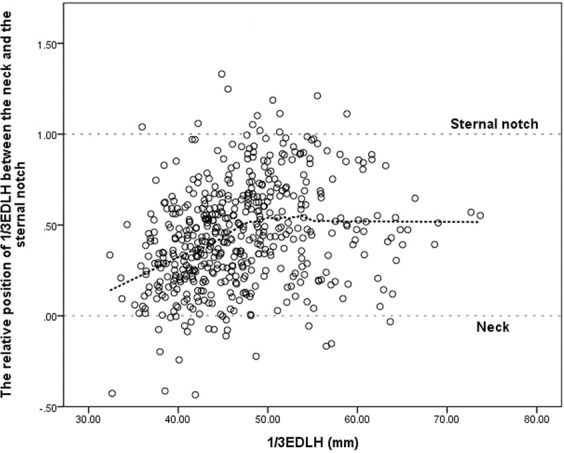
Relative locations corresponding to one-third anteroposterior diameter at the lower half level of the sternum between the neck and the sternal notch. EDLH: external anteroposterior diameter of the chest at the lower half of the sternum.

Figure 2 shows the relative locations corresponding to one-third external AP diameter at the lower half of the sternum between the neck and sternal notch. The LOESS curve shows that the location corresponding to one-third external AP diameter at the lower half of the sternum is closer to the neck when one-third external AP diameter at the lower half of the sternum is smaller, gradually getting closer to the mid-landmark as one-third external AP diameter at the lower half of the sternum increases, plateauing at approximately 50 mm.

## Discussion

The results of this study showed that the neck and sternal notch, the two anatomical landmarks of CC depth in children were not close to one-third AP depth at the lower half of the sternum. Instead, in most subjects, the depth corresponding to one-third of the AP chest diameter was located between the two landmarks.

Since 2005, the ERC recommended a target CC depth of one-third the AP chest diameter for CPR in children^2^. In 2010, the AHA also recommended the same target CC depth for children^1,6^. Meanwhile, several studies have reported that the recommended CC depth is not well achieved. Van Tulder *et al*. observed that two-thirds of professional and lay rescuers failed to make correct visual estimates of the recommended CC depth on the horizontal axis^7^. In another study, one-third of lay rescuers failed to achieve the target CC depth, even those in the professional health care group. A study on paediatric resuscitation also reported that 74% of professional rescuers failed to reach the target CC depth recommended by paediatric guidelines^3^. A recent multicentre study demonstrated similar poor-quality CPR during simulated paediatric cardiac arrest^8^. Based on our results and those of a previous study, the application of anatomical landmarks as resuscitation aids in paediatric CPR may increase the probability of achieving the target CC depth. The anatomical landmarks presented in our study have been used in several clinical situations. We identified the sternal notch as a landmark by means of central venous catheterisation. In CPR situations, the sternal notch is used to determine the location for CC. Moreover, we are accustomed to the use of the neck as the anatomical landmark, e.g. checking for carotid artery pulsations in the neck, when performing CPR.

The results of the present study suggest that it is not reasonable to use the two landmarks independently in paediatric CPR. The average depth of each landmark differed from the average one-third AP depth at the lower half of the sternum. Rather, the average depth corresponding to the mid-point of the two landmarks was close to one-third AP depth at the lower half of the sternum. Thus, using the neck alone as a landmark can lead to deeper CC than when using one-third AP depth of the chest. Conversely, when the sternal notch is used separately, it can lead to shallow CC. In a recent study on landmarks for CC depth in adults, Kim *et al*. reported that the sternal notch can be a landmark for CC depth and that its depth is close to one-fourth of the AP chest diameter^4^. However, paediatric guidelines recommend one-third AP depth, which is greater than the depth for adults. Because of the size difference in the fraction number, in our study, which consisted of children, one-third AP depth was deeper than the sternal notch depth.

The thorax of children, aged one-to-nine years, transforms from a circular cross-section to an ovoid shape, whereas the volume of the chest rapidly increases in an upslope pattern^5^. The rate of increase in the volume of the chest before the age of five years is greater than that thereafter. Unlike thorax growth, the circumference of the neck gradually increases until adolescence^9^. In Fig. 2, we observed a trend in the relative locations corresponding to one-third external AP diameter at the lower half of the sternum between the neck and the sternal notch according to chest size. Because of the difference in the ontogenetic growth of the neck and chest, one-third AP depth of the chest might be close to the depth of the neck for small chest sizes and close to the sternal notch for large chest sizes.

In the current study, although the position corresponding to one-third AP depth of the chest differed slightly between the two landmarks according to chest size, its deviation from the space between the 2 landmarks was less than 10%. Sutton *et al*. recently reported that deep CC was associated with increased survival in children older than one year of age^10^. However, a CC that is too deep might lead to patient injury; therefore, balancing these factors is important to achieve the best outcome^11–13^. The 2015 AHA guidelines introduced an upper limit of CC depth for adults, beyond which the outcomes could be adverse^14,15^. In our study, CC depths beyond the space between the two landmarks were either too shallow or too deep. The developmental trends in children and the results of this study suggest that the neck can be used as a landmark for upper limitations to prevent CCs deeper than required in children of all ages. On the contrary, CCs reaching below the depth of the sternal notch in all paediatric age groups will result in very shallow CCs. In other words, the sternal notch may be used as a landmark for lower limitations. Additionally, considering that the mid-point of the two landmarks was closer to the depth recommended by the paediatric guidelines, it is likely that both landmarks can be used as indicators for upper and lower limits. According to these results, the space between the two landmarks may be appropriate as an anatomical landmark for CC depth. It seems reasonable to use a combination of these landmarks rather than only one landmark to determine the appropriate CC depth.

Recently, real-time audio-visual feedback devices have been widely used in clinical practice and training^16^. Although the number of studies showing improved clinical outcomes using such devices is limited, feedback devices could help achieve the CPR metrics indicated in the guidelines^17^. With regard to the CC depth for children, the major guidelines, ERC and AHA, recommend fractional CC depth^1,2,6^. To apply this fractional CC depth in paediatric CPR, the chest size of each child should be determined in each CPR event since the size can differ for each child. Even if feedback devices are used with children, the chest AP diameter must be measured to individualise the target CC depth for each child. In the pre-hospital environment, where there are no feedback devices or rulers, the rescuer (especially lay rescuers) must visually estimate one-third of the AP chest diameter. In all circumstances where no feedback device is available, alternative systems using anatomical landmarks may help achieve the CC depth recommended in the guidelines. A feedback device may be easy to use when the absolute value of compression depth is applied, such as ‘4 cm for infant and 5 cm for children as indicated in the AHA guidelines’^1^. However, the absolute value of the compression depth applied for children may be too deep for younger children^13,15,18^. Therefore, the inherent physiologic landmark can be utilised.

In the present study, the average difference in the depth between the 2 landmarks was 15.9 mm, which is wider than the difference between the upper and lower thresholds of adult CC depth, i.e. 10 mm^14,19^. However, the evidences supporting this threshold in the guidelines for adults are insufficient. Moreover, there is a dearth of studies on the upper limits for children^18^. The boundary between two landmarks cannot be completely distinguished in real clinical practice, as measured via CT image in this study. Meanwhile, the anatomical landmarks used to determine the hand placement during CC have been utilised in clinical practice, but changes have been made in several guidelines^1,2,20^. The anatomical landmarks for the hand placement during CC recommended in the latest guidelines cannot be considered optimal for every patient. Therefore, the anatomical landmarks presented in this study may be used as CPR aid, using only the approximate values, not the exact ones. In clinical settings, it is difficult to visually estimate the extent of the depth of 10 mm than that of 15 mm. Gregson *et al*. reported that it is difficult to control CC force in simulation studies despite the use of audio-visual feedback devices^21^. The narrower the target space used as a landmark the more limited it may be. However, to the best of our knowledge, no study has addressed this topic and its importance for CPR in children. Hence, further studies are needed to evaluate the findings of the present study in clinical practice.

This study has some limitations. First, it was retrospective and observational in nature and was conducted in a single centre. Thus, even if the exclusion criteria were strictly applied, there is a possibility of selection bias. Second, situations based on CT images were not taken into consideration during actual CPR situations. Third, because the study population comprised paediatric patients, the respiratory phases were not constant during the CT scan because of the difficulty in controlling their breathing. The depth of the neck may be constant between different respiratory phases. The sternum may be relatively fixed superiorly at the thoracic inlet including the sternal notch, moving predominantly at the lower part of the sternum during respiration^4,22^. However, in children, the sternal notch may also be influenced by respiration because chest compliance varies greatly with age. Fourth, although the overall sample size was large, the number of participants in each age group may have been too small. Hence, the findings cannot be generalised. Fifth, the population of our study comprised only Korean children. The Korean national growth chart for the chest wall diameter has not yet been reported. There are also no data available to compare thoracic measurements in relation to other demographic groups. However, the thoracic dimensions may differ, considering that the height and weight vary among different national and ethnic groups^23^. This may also be the reason why the results of this study cannot be generalised. Lastly, we investigated the theoretical possibility of using landmarks to guide compression depth in this study, but the results cannot support the utility of landmarks in a clinical setting. To confirm their utility, manikin simulation studies and further empirical studies involving other population groups may be needed.

In conclusion, the sternal notch and neck are inappropriate landmarks to guide compression depth in paediatric CPR. However, CC depths beyond the space between the two landmarks may be either too shallow or too deep. Therefore, further studies are needed to determine their efficacy.

## Methods

This retrospective study was conducted at a regional tertiary emergency centre in South Korea with an annual admission of 22,000 paediatric patients. It was approved by Samsung Changwon Hospital’s Institutional Review Board (SCMC 2017-12-081). Owing to the retrospective nature of the study, the need for informed consent was waived

### Patient enrolment and materials

From January 2005 to August 2017, paediatric patients aged 1–9 years, who underwent a chest CT scan at the hospital, were enrolled. Patients with chest deformities such as pectus excavatum (funnel chest) or pectus carinatum (pigeon chest), status asthmaticus, Poland syndrome, sternal defects, and thoracic ectopia cordis; those with a history of neck or chest surgery and whose external diameter could not be measured because of massive subcutaneous emphysema in the neck or thorax; those with skin inflammation around the neck or thorax; and those who had a tumour or a history of tumour around the landmarks were excluded. Patients in whom the landmarks could not be identified were also excluded. The CT scans were generated using a Brilliance 16 CT device (Philips Healthcare, Andover, MA). A picture archiving and communication system (Marotech, Seoul, South Korea) was used to analyse the images.

### Definitions of measurements

**Figure 3:**
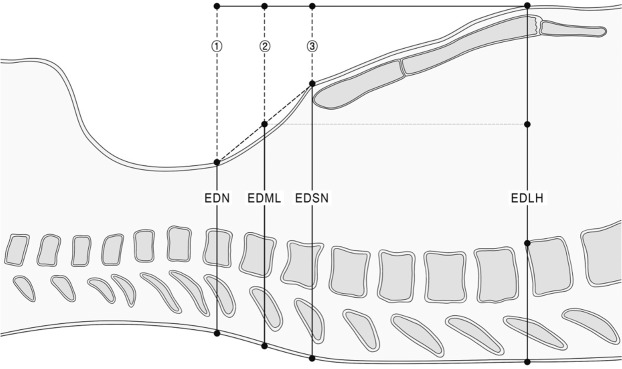
Simplified schematic presentation to measure the external diameter and depth of each landmark. 1. Neck depth. 2. Mid-landmark depth. 3. Sternal notch depth. EDN: external anteroposterior diameter of the neck. EDML: external anteroposterior diameter of the chest at the mid-point between the two landmarks. EDSN: external anteroposterior diameter of the chest at the sternal notch. EDLH: external anteroposterior diameter of the chest at the lower half of the sternum. Jung-yon Ko granted permission to use this illustration.

**Figure 4:**
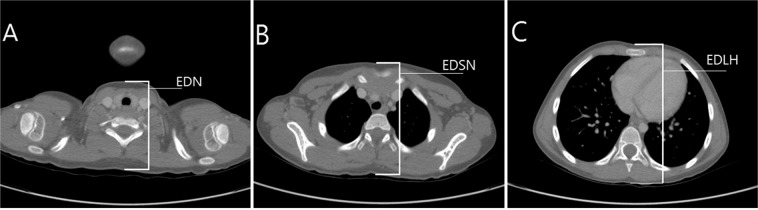
Axial image of the neck (**A**), sternal notch level (**B**), and at the lower half level of the sternum (**C**) from a computed tomography scan and the method for measuring each external anteroposterior diameter. EDN: external anteroposterior diameter of the neck. EDSN: external anteroposterior diameter of the chest at the sternal notch. EDLH: external anteroposterior diameter of the chest at the lower half of the sternum.

We evaluated two anatomical locations, the anterior surfaces of the neck and the sternal notch, as the landmarks for CC depth in children and the mid-point between the two locations. We defined the location of the anterior surface of the neck as the flattened plane between the sternal notch and thyroid cartilage. The criterion for CC depth recommended by the guidelines was defined as one-third of the external AP diameter at the lower half of the sternum. The external AP diameter of the neck, the chest diameter at the level of the sternal notch, and the level of the lower half of the sternum were measured (Fig. 3). The external AP diameter of the neck was measured from an axial slice at the upper border level of the seventh cervical vertebral body. The external AP diameter at the lower half of the sternum was measured from an axial slice at a level one-quarter from the xiphoid process identified by levelling the total sternal length in the midline sagittal and coronal views. The external AP diameter at the sternal notch was measured from an axial slice at the upper border level of the manubrium. The external diameter was calculated by measuring the length of a line drawn perpendicularly from the skin of the bone or the cartilage in each level anteriorly to the skin of the spinous process of the vertebra posteriorly (Fig. 4).

To estimate the depths of the landmarks from a virtual point at the same height as the position for the CC, we calculated the differences between the external AP diameters of the neck, sternal notch, and mid-landmark and the lower half of the sternum. The difference between the external AP diameter at the lower half of the sternum and the external AP diameter at the sternal notch was defined as the depth of the sternal notch (Fig. 1). In addition, the difference between the external AP diameter at the lower half of the sternum and the external AP diameter of the neck was defined as the depth of the neck. The external AP diameter at the mid-point between the two landmarks was calculated by averaging the external AP diameters of both landmarks and then calculating the difference between the mean obtained and external AP diameter at the lower half of the sternum. The difference between the external AP diameter at the mid-point and external AP diameter at the lower half of the sternum was defined as the depth of the mid-landmark. We compared the depth of each landmark to one-third of the AP diameter of the chest at the lower half of the sternum, which is the CC depth recommended by current paediatric CPR guidelines. A landmark with a depth closer to one-third of the AP diameter of the chest at the lower half of the sternum suggested that that location was appropriate for being considered a landmark for CC depth.

CT measurements for each subject were conducted by 2 radiologists who previously reviewed the CTs of 30 randomly selected subjects to standardise their measurements. If the measurement differences between the 2 raters exceeded 5 mm, the differences were resolved by re-assessment. Data analysis was performed by averaging the measurements obtained by the 2 raters. The inter-rater reliability of the measurements between the 2 raters was assessed by intra-class correlation coefficient (r) (r = 0.914, 0.905, and 0.968 for external AP diameter of the neck, chest diameter at the level of the sternal notch, and level of the lower half of the sternum, respectively).

### Statistical analyses

Continuous variables were presented as means ± standard deviation (SD). Categorical variables were expressed as numbers (percentage). Pearson’s chi-square test was conducted to analyse categorical variables. Analyses of variance were carried out to analyse the variables in each age group. One-sample *t*-tests were conducted to compare the depth of each landmark and one-third AP diameter of the chest. The relationship between the depth of each landmark and one-third AP diameter of the chest at the lower half were analysed using Bland–Altman plots. All reported P-values were two-sided and P-values < 0.05 were considered statistically significant. IBM SPSS Statistics version 24.0 (IBM Corp., NY, USA) was used for all statistical analyses.

We plotted a scatter plot of the relative locations of one-third external AP diameter at the lower half of the sternum between the neck and sternal notch. The position corresponding to one-third external AP diameter at the lower half of the sternum was calculated from the difference between the depth of the neck and one-third external AP diameter at the lower half of the sternum; then, the relative position value was obtained by calculating its ratio to the difference between the depth of the neck and sternal notch.

## Data Availability

The datasets generated and analysed during the current study are available from the corresponding author on reasonable request.
